# The Effect of Pelvic Muscle Exercises on Urinary Incontinency and Self-Esteem of Elderly Females With Stress Urinary Incontinency, 2013

**DOI:** 10.5539/gjhs.v7n2p71

**Published:** 2014-09-28

**Authors:** Marzieh Kargar Jahromi, Malihe Talebizadeh, Maryam Mirzaei

**Affiliations:** 1 Community Health Nursing, Faculty Member, Jahrom University of Medical Science, Jahrom, Iran; 2 BSc of Nursing, Jahrom University of Medical Science, Jahrom, Iran; 3 Critical Care Nursing, Faculty Member, Jahrom University of Medical Science, Jahrom, Iran

**Keywords:** pelvic muscle exercises, urinary incontinency, self- esteem, elderly

## Abstract

**Introduction:**

Millions of women are afflicted with stress urinary incontinence. Urinary incontinence is mentioned as one of the geriatric syndromes, together with pressure ulcers, functional decline, falls, and low self-esteem. The aim of the present study was to determine the effect of pelvic muscle exercises on urinary incontinency and self- esteem of elderly females with stress urinary incontinency in Shiraz, Iran, 2013.

**Material and Method:**

In this interventional study, 50 old females aged 60-74 years were chosen among the members of Jahandidegan center, and they were asked to sign the informed consent form and complete the demographic questionnaire. Then, Quid questionnaire was used for choosing the type of incontinence in the elderly females. Next, the participants completed the ICIQ and self-esteem questionnaires. Then, they were randomly assigned to case and control groups. Each participant took part in 8 training classes. Finally, the subjects filled the ICIQ and self-esteem questionnaires before and 2 months after the intervention.

**Result:**

The results is shown that after the intervention, ICIQ score has a significant difference between the two groups (P=0.001). Also, after the treatment, self-esteem average scores of studied unit indicated a significant statistical difference in experimental group. In other words, the training sessions improved the score of self-esteem in the experimental group (P<0.001) versus control group (P=0.08).

**Conclusion:**

Pelvic muscle exercises were an empowerment mechanism for incontinent women in improving their quality of life and self-esteem, so recommended that such these exercising programs be used in elderly health care centers as a factor to improve health promotion of elderlies ’that are suffering from urinary incontinence.

## 1. Introduction

As population ages, the number of patients presenting to their primary care physicians with urologic problems are significantly increasing. Urologic issues are the third most common type of complaints in patients 65 year or older, accounting for at least a part of 47% of office visits (Dyche & Hollander, 2009). Urological symptoms are the major public health problems in the USA (Ho, Chan, Woo, Chong, & Sham, 2009). One of the most predominant urologic problems among the elderly is urinary incontinence (Dyche & Hollander, 2009). The International Continence Society (ICS) Standardization Committee defined urinary incontinence as “a condition in which involuntary loss of urine is a social or hygienic problem and is objectively demonstrable (Mons, Chartier-Kastler, Hampel, Samsioe, & Hunskaar, 2007; Paick, Kim, Oh, & Ku, 2007).

Millions of women are afflicted with stress urinary incontinence (SUI). Stress urinary incontinence is involuntary loss of urine with sneezing, coughing and effort and is a frequent and bothersome symptom that is common in the elderly population (GHodsbin, Kargar, Jahanbin, & Sagheb, 2012).

Urinary incontinence (UI) impacts an estimated 15 to 35% of the adult ambulatory population 60 and older that live in the community with prevalence rates for women being twice that of men (Thom, 2000). Urinary incontinence is a common condition with important social and psychological consequences (Bogner, Gallo, Swartz, & Ford, 2002).

Involuntary loss of urine has multiple implications for the sufferer. Incontinence also has been noted to be a major barrier to social interests, entertainment, or physical recreation (Shelton Broome, 2003). Significant urinary incontinence may cause shame and lead to withdrawal from social activities. Elderly outpatients describe their experience with incontinence as embarrassing, upsetting, and distressing (Bogner, Gallo, Swartz, & Ford, 2002). Persons with urinary incontinence may be anxious about not having ready access to a toilet and may worry about the possibility of a urinary accident in public (Ghodsbin, Kargar, Jahanbin, Sagheb, & Keshavarzi, 2012).

In the literature, UI is mentioned as one of the geriatric syndromes, together with pressure ulcers, functional decline, falls, and low self-esteem. Depression and low self-esteem have been suggested to co-occur in incontinent persons (Ruby, Hanlon, & Fillenbaum, 2005).

Previous studies in community-dwelling older people have suggested that the disability to perform activities of daily living (ADL) may be associated with new onset urinary incontinence in the older population (Coll-Planas, Denkinger, & Nikolaus, 2008; Jenkins, & Fultz, 2005). Independency in ADL might cause reduced quality of life, self-esteem and social isolation (KO, Lin, & Salmon, 2005).

Current treatments for UI include behavioral (e.g., bladder training, fluid manipulation, scheduled toileting, pelvic floor muscle exercises (Sampselle, 2003). Behavioral techniques are now currently recommended as first-line therapy in the treatment of UI (Marcell, Ransel, Schiau, & Duffy, 2003). Behavioral interventions are usually relatively inexpensive and easy to implement, but their effectiveness depends chiefly on patient motivation and compliance (Dickson, 2008).

The use of pelvic floor muscle (PFM) exercises in the treatment of stress urinary incontinence is based on two functions of the pelvic floor muscles: support of the pelvic organs, and a contribution to the sphincter closure mechanism of the urethra (Kumari, Jain, Mandal, & Singh, 2008). A PFM program may be prescribed to increase strength, endurance, and coordination of muscle activity. Strength training decreases the frequency of SUI with time, and skill training immediately reduces the amount of leakage (Hay-Smith & Dumoulin, 2006). Pelvic floor rehabilitation is generally the first-line treatment for female patients with SUI (Jundt, Peschers, & Dimpfl, 2002).

The aim of the present study was to determine the effect of Pelvic floor muscle exercises on urinary incontinency and Self-Steam of elderly females with stress urinary incontinency, referring to jahandidegan center in Shiraz, Iran, 2013.

## 2. Methods and Materials

### 2.1 Setting

Jahandidegan center is a day-time center for older adults located in Kholdebarin Park in Shiraz, Iran.

### 2.2 Data Collection

The instruments used for the study were the Questionnaire for urinary incontinence diagnoses (QUID), International Consultation on Incontinence Questionnaire (ICIQ) and self-esteem questionnaires. Quid questionnaire was used for choosing the type of incontinence in the elderly females. Given the importance of urinary incontinence on quality of life, there is increasing interest in the use of well-constructed questionnaires. Stress and urge urinary incontinence, the most common conditions that cause female urinary incontinence symptoms, has different pathophysiologic mechanisms, epidemiologic characteristics, and treatments. Distinguishing between these types of urinary incontinence is important in clinical practice and for research purposes.

On the basis of previous research and expert clinical opinion, we used a QUID questionnaire to distinguish stress from urge incontinence that includes 6 questions and requires about 5 minute to complete. We believe that this is partly because national guidelines recommend an extended evaluation for classifying type of incontinence that is not practical in health care centers. The QUID questionnaire is a simple, quick, and reproducible test with acceptable accuracy for classifying urge, mix and stress incontinence among women who are appropriate for evaluation and treatment in health care centers.

In Ghodsbin’s study, a preliminary pilot study was carried out to determine the validity and reliability of Quid questionnaire for Iranian elderly. The original questionnaire was translated into Persian by three professors of Nursing and Midwifery College in Shiraz University of Medical Sciences, and then it was back translated from Persian into English. In the next step, as approved by Shiraz Welfare Organization, 25 females aged 60–74 years were chosen from Shiraz Jahandidegan Center to fill out the questionnaire twice with three weeks interval. Statistical analysis showed that the Cronbach α coefficient of the Quid questionnaire was 0.86 and the performed test-retest had an appropriate reliability (Ghodsbin, Kargar, Jahanbin, Sagheb, & Keshavarzi, 2012). In other study internal consistency and test-retest reliability estimates were good. Sensitivity and specificity were 85% (95% CI, 75%, 91%) and 71% (95% CI, 51%, 87%), respectively, for stress urinary incontinence and 79% (95% CI, 69%, 86%) and 79% (95% CI, 54%, 94%), respectively, for urge urinary incontinence (Bradley, Rovner, Morgan, Berlin, & Novi, 2005).

The second questionnaire includes the standard ICIQ questionnaire. It is a simple and brief questionnaire that can be self-administered contains 6 questions that the first two questions related to demographic variables and the next four questions related to urinary incontinence conditions. Sum of the questions’ scores 3 to 5 is the questionnaire average scores.

The third questionnaire was Rosenberg’s self-esteem evaluation. Rosenberg’s evaluation is a standard evaluation that includes 10 sentences or comments that shows the real feeling of each person about each sentence in one of four options: very agree, agree, disagree and very disagree determined by a cross in front of each sentence with 1 to 4 points and the total point is obtained by summing up the points for 10 questions. Thus, 10 and 40 points indicate the minimum and maximum of self-esteem.

### 2.3 Intervention

In this interventional study, 50 old females aged 60–74 years were chosen among the members of Jahandidegan center, and they were asked to sign the informed consent form and complete the demographic questionnaire. Then, Quid questionnaire was used for choosing the type of incontinence in the elderly females. Next, the participants completed the ICIQ and self-esteem questionnaires. The inclusion criteria were age 60–74 years, having Quid score for incontinence type (stress score ≥ 4, clinical symptoms of urinary incontinence within the last 6 months, and willing to participate in the study. The exclusion criteria were absence in more than two training sessions, suffering from central nervous system disease (e.g. multiple sclerosis, cerebrovascular accident or acute mental illness and dementia, recent urology surgery (for less than three months), history of genitourinary malignancy, current urinary infection, hysterectomy and diabetic mellitus. Then, they were randomly assigned to case and control groups. Each participant took part in 8 training classes. Participants were taught about the anatomy of the pelvic floor and lower urinary tract, physiology, and continence mechanisms by the trained nurse. All were taught to contract the pelvic floor muscles correctly. Participants were asked to conduct 8-12 high intensity (close to maximum) contractions three times a day at home with additional training in groups once a week for 45 minutes. Group training was performed in lying, standing and sitting positions with legs apart to emphasize specific strength training of the pelvic floor muscles and relaxation of other pelvic muscles. Participants aimed at holding each muscle contraction for 6-8 seconds, three or four fast contractions were then added. The rest period was about 6 seconds. A total of 8 to 12 contractions were completed in each position with maximal contraction effort encouraged. Body awareness, breathing, relaxation exercises, and strength training for the abdominal, back, and thigh muscles were performed to music between positions. The participants were encouraged to use their preferred position and perform equally intensive contractions at home. Finally, the subjects filled the ICIQ and self-esteem questionnaires before and 2 months after the intervention.

### 2.4 Data Analysis

The results were analyzed by SPSS version 16. The data were examined using percent, mean and standard deviation and independent t-test.

## 3. Result

**Figure 1:**
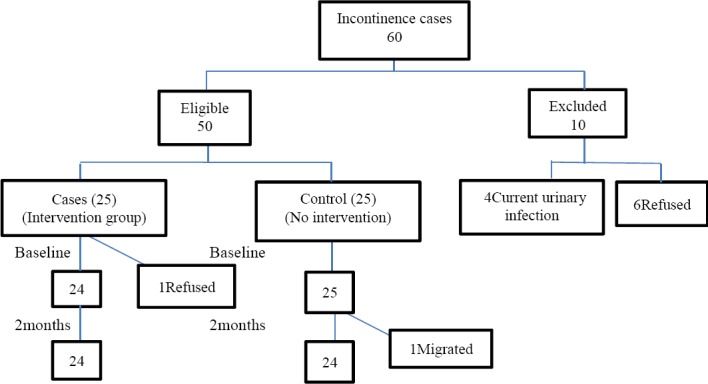
Participant flow through the study

Of the 60 women with urinary incontinence, 10 (16.7%) were excluded from the randomized controlled trial leaving 50 for randomization into the 2 groups. At the onset of the study, 1 woman in the study group refused; and 1 woman in the control group was lost to follow up during the trial (Figure 1).

In the present study, the participants were examined for urinary incontinence and self-esteem conditions in two experimental and control groups. The average age of studied samples in control group was 68.05± 9.10 and in experimental group was 67.15±8.36. The average duration of urinary incontinence in the experimental group was 5.1±2.3 and in the control group 4.1±2.6 years that it wasn’t observed a statistically significant difference between two groups through using the statistical t-Test exam. Also, in other terms of demographic information, there was no difference between two groups and they were completely the same.

In assessing of the urinary incontinence, 5 parameters were studied that include the following:

**1. The number of frequencies of leakage urine:** According to findings in experimental and control group, before and after the treatment, a significantly statistical difference was observed in the number of urinary incontinence frequencies. (The experimental group: p = 0.001 and the control group: p = 0.002). In comparison, before the treatment, there was no difference between two groups (p = 0.2) but after the treatment was observed a significant statistical difference (p = 0.04). Therefore, it means that using the provided training made the experimental group be improved.
**2. The measurement of amount of leakage urine:** Based on findings in experimental and control group, before and after the treatment, a significant statistical difference was observed. (The experimental group: p = 0.001 and the control group: p = 0.003). In comparison, before the treatment, there was no difference between two groups (p = 0.7). But after the treatment a significant statistical difference (p = 0.001) was observed.
**3. The impact of urinary incontinence on quality of life:** Based on findings in experimental and control group, before and after the treatment, a significant statistical difference was observed. (The experimental group: p = 0.04 and the control group: p = 0.01). In comparison, before doing the treatment, the impact of urinary incontinence on life quality was no different between two groups (p = 0.1) but after the treatment a significant statistical difference (p = 0.01) was observed.
**4. Time of leakage urine:** According to findings a significantly statistical difference wasn’t observed before and after the treatment in both groups. (The experimental group: p = 0.9 and the control group: p = 0.4). And also between the two groups wasn’t observed any differences; before the treatment (p = 0.1) and after it (p = 0.6).
**5. Total scores of questionnaire:** As shown in Table 1, the mean score for ICIQ questionnaire before the intervention in the two groups is almost the same (P = 0.3). The results is shown that after the intervention, ICIQ score has a significant difference between the two groups (P = 0.001). In other words, the training sessions improved the score in the experimental group versus control group.

**Table 1:** Results of the study according to ICIQ questionnaires

Group Time variable	Case		Control		P-value Before Intervention	P-value After Intervention
	
	Before	2m later	Before	2m later		
	
	N Percent	N Percent	N Percent	N Percent		
How often do you leak urine?						
(1)About once a week or less often	3(10)	16(53.3)	0(0)	2(6.7)		
(2)Two or three times a week	6(20)	3(10)	1(3.3)	4(13.3)	P=0.2	P=0.04
(3)About once a day	2(6.7)	10(33.3)	3(10)	20(66.7)		
(4)Several times a day	18(60)	1(3.3)	24(80)	3(10)		
(5)All the time	1(3.3)	0(0)	2(6.7)	1(3.3)		
We would like to know how much urine you think leaks. How much urine do you usually leak (whether you wear protection or not)?						
(1) None or A small amount	15(50)	24(80)	10(33.3)	9(30)	P=0.7	P=0.001
(2) A moderate amount	10(33.3)	4(13.3)	5(16.7)	11(36.7)		
(3)A large amount	5(16.7)	2(6.7)	15(50)	10(33.3)		
Overall, how much does leaking urine interfere with your everyday life? Please ring a number between 0 (not at all) and 10 (a great deal)						
(1-3) mild	3(10)	12(40)	2(6.7)	3(10)	P=0.1	P=0.01
(4-6) moderate	14(46.7)	15(50)	9(30)	12(40)		
(7-9) severe	9(30)	2(6.7)	14(46.7)	15(50)		
(10) very severe	4(13.3)	1(3.3)	5(16.7)	0(0)		
When does urine leak? (Please tick all that apply to you)						
(1) Never – urine does not leak or Leaks before you can get to the toilet	15(50)	15(50)	14(46.7)	14(46.7)		
(2) Leaks when you cough or sneeze	12(40)	12(40)	12(40)	12(40)		
(3) Leaks when you are physically active/exercising and sleeping	1(3.3)	1(3.3)	0(0)	0(0)		
(4) Leaks when you have finished urinating and are dressed and Leaks all the time	2(6.7)	2(6.7)	4(13.3)	4(13.3)		
Sum of Scores	10.78 ± (3.20)	9/07 ±(2.33)	13.93)±4.2)	12.30 ±(3.6)	P=0.3	P=0.001

**Table 2:** Comparison of mean of self-esteem score before and after the intervention in the two groups

P- value	After	Before	Time Group
**0.001**	27.66±4	21.50±4.21	**Case**
**0.08**	22.38±5.04	22.88± 4.75	**Control**

In the present study, there was no significant difference between two experimental and control groups before the treatment in self-esteem scores. But after the treatment, self-esteem average scores of studied unit indicated a significant statistical difference in experimental group. In other words, the training sessions improved the score of self-esteem in the experimental group (P<0.001) versus control group (P = 0.08).

## 4. Discussion

In investigating of urinary incontinence in 5 mentioned parameters, some changes were observed in two groups before and after the treatment. However, regarding to issues such as the women’s lifestyle in our society, from researcher point of view in Iran women’s society, and with considering the style of their living such as washing dishes and clothes in sitting position, using Iranian toilet, improper diet and etc. can be part of factors to influence more on these behavioral interferences.

In a community based study that was done by Sharon et al. in Boston of Spain with the aim of the severity impact of micturition dribble on life quality of 3202 women and 2301 men with ages 30-79, 30 percent of women and 18 percent of men reported the micturition dribble that they were mostly mild to moderate. Women reported more severe micturition dribble than men (1.5 percent in contrast of 0.9 percent). Analyzing of some variables represented that simultaneously by increasing the intensity of micturition dribble; the life quality score is associated with more stagnation. This study showed that micturition dribble causes the disruption of life quality in both women and men (Sharon et al., 2010).

In a semi experimental study was done by Seyed Rasooly et al. in Tabriz (Iran), on 60 elderlies with the aim of applying principles of the evidence-based nursing care for urinary incontinence. The findings in studied samples of experimental and control group had a meaningful statistical difference in micturition dribble frequencies, micturition dribble rate, and the impact of micturition dribble rate on life quality before and after the treatment (p = 0.001) (Syed Rasouli, Valizadeh, & Haj Ebrahim, 2011). It included that incontinence makes a significant impact on a woman’s Quality of Life (QOL). In our study on domain of the impact of urinary incontinence on quality of life a significant statistical difference was observed in experimental and control group before and after the treatment.

In a study that was done by Shah Ali at Shahid Akbar Abady hospital with the aim of the effect of kegel exercises on urinary incontinence, 50 women with ages between 25-54 who were suffering urinary incontinence and were qualified to enter the study, the results represented that the average score of life quality of urinary incontinence suffering women was 53.15 before doing kegel exercises and after the treatment was 73.82 that there was a significant difference between them (p=0.0001) (Shah Ali, Kashanian, & Azari, 2011)

In a study of Godey et al. in The United States, 2004, with the title of the effect of behavioral and medication interferences on urinary incontinence cure, two methods, the kegel muscles exercises and medication were compared for 3 months. The studied samples of women and men were 40 to 60 years old that were in two groups experimental and control (each group 15 persons). In experimental group the kegel exercises were taught face to face that lasted for 10 weeks and in control group two medicines Botanicol and Oxybotin were used and the frequencies of urinary incontinence, micturition dribble rate and the impact of treatment on the life quality of both groups were checked before and after the treatment. Applied tools were ICIQ form and SF-38 standard questionnaire. By considering the frequencies of urinary incontinence, in experimental group, 73 percent of samples, their frequencies reduced from 5 times in a day to twice in a day and in control group, 63 percent of samples reduced from 5 times a day into 3 times in a day. The micturition dribble rate in 82 percent of experimental group and 56 percent of control group was reported a little bit or never. Considerable changes happened in physical and spiritual dimensions of the life quality of experimental group and were associated with improvement of their social interactions. Recent study shows that behavioral therapy with biofeedback or without it has useful clinical results and behavioral therapy should be used in a clinical environment in advanced (Goode, Burgio, Kenton, Litman, & Richter, 2011). Our findings are consistent with these studies. The frequencies of leakage urine and amount of leakage urine in experimental group were reduced after the intervention. It indicated that urinary incontinence is significantly improved by behavioral interventions.

In a study that was done on 30 hospitalized patients in Imam Reza and Gharazi hospitals of Sirjan city in Iran with the aim of the kegel exercises effect on urinary incontinence cure by Khalili and Mohammadi the results indicated that during 3 months the urinary incontinence frequencies reduced to less than 30 percent and kegel exercises can be used as an efficient method to cure the urinary incontinence (Baba Mohammadi & Khalili, 2006).

The result of our study showed the significant impact of exercises on urinary incontinence and increasing self-esteem of elderly females that is consistent to the results of some researches in this field. One possible explanation is that having SUI causes problems with self-esteem and doing kegel muscles exercises leads to increase the elderly’s life quality and enhance their self-esteem, for example McAuley and Elavssky reported that there is a positive correlation between the physical activities rate and accuracy of doing exercises with self-efficiency and self-esteem in elderlies (McAuley & Elavssky, 2005). Other study showed, although there’s no relationship between exercises rate, body satisfactory, and self-esteem among youths, but doing motivating sport activities, improve health and physical capabilities and increase self-esteem in elderlies (Tiggemann & Williamson, 2000).

In a study that was done by Thomas in England a group which attended in the aerobic exercises for 8 weeks the average scores of self-esteem had increased after the treatment (Thomas, 1999). Walter et al. in his study found out a regular exercising plan with moderate intense causes positive changes and enhance self-esteem (Walters & Martin, 2000). Our study extends this body of research which emphasizes the important psychosocial and physical health consequences of kegel muscles exercises on self-esteem and QOL of elderly females with SUI.

Limitations of the study were as fallowing:

- Not doing the important points about lifestyle can put the treatment and their advantages under a question.
- Recognizing kegel muscles correctly and doing an effective contraction is a key point in determining of a useful treatment and in a case that the studied samples be not able to recognize the muscles, not only will it have positive effect but it will also a negative effect on urinary incontinence if abdomen muscles be contracted.

## 5. Conclusion

Pelvic Muscle Exercises were an empowerment mechanism for incontinent women in improving their quality of life and self-esteem, so recommended that such these exercising programs be used in elderly health care centers as a factor to improve health promotion of elderlies ’that are suffering from urinary incontinence.
